# High systemic IL-6 is associated with worse prognosis in patients with non-small cell lung cancer

**DOI:** 10.1371/journal.pone.0181125

**Published:** 2017-07-17

**Authors:** Estela Maria Silva, Vânia Sammartino Mariano, Paula Roberta Aguiar Pastrez, Miguel Cordoba Pinto, António Gil Castro, Kari Juhani Syrjanen, Adhemar Longatto-Filho

**Affiliations:** 1 Teaching and Research Institute, Barretos Cancer Hospital–Pio XII Foundation, Barretos, Sao Paulo, Brazil; 2 Department of Chest, Barretos Cancer Hospital–Pio XII Foundation, Barretos, Sao Paulo, Brazil; 3 Research Institute of Life and Health Sciences (ICVS), University of Minho, Braga, Portugal; 4 ICVS / 3B's—Associated Laboratory to the Government of Portugal, Braga / Guimarães, Portugal; 5 Department of Clinical Research—Biohit Oyj, Helsinki, Finland; 6 Medical Laboratory of Medical Investigation (LIM) 14, Department of Pathology, Faculty of Medicine, University of São Paulo, Sao Paulo, Sao Paulo, Brazil; University of South Alabama Mitchell Cancer Institute, UNITED STATES

## Abstract

Characteristic cytokine patterns have been described in different cancer patients and they are related to their diagnosis, prognosis, prediction of treatment responses and survival. A panel of cytokines was evaluated in the plasma of non-small cell lung cancer (NSCLC) patients and healthy controls to investigate their profile and relationship with clinical characteristics and overall survival. The case-controlled cross-sectional study design recruited 77 patients with confirmed diagnosis of NSCLC (cases) and 91 healthy subjects (controls) aimed to examine peripheral pro-inflammatory and anti-inflammatory cytokines (IL-2, IL-4, IL-6, IL-10, IL-17A, TNF and IFN-γ) by Cytometry Beads Arrays (CBA Flex) in. The cytokine IL-6 showed a statistically significant difference among groups with increased expression in the case group (p < 0.001). The correlation between the cytokines expression with patient’s clinical characteristics variables revealed the cytokine IL-6 was found to be associated with gender, showing higher levels in male (p = 0.036), whereas IL-17A levels were associated with TNM stage, being higher in III–IV stages (p = 0.044). We observed worse overall survival for individuals with high levels of IL-6 when compared to those with low levels of this cytokine in 6, 12 and 24 months. Further studies of IL-6 levels in independent cohort could clarify the real role of IL-6 as an independent marker of prognostic of NSCLC.

## Introduction

Lung cancer is the leading cause of cancer-related deaths worldwide [1]. Histologically, lung cancer is divided into two main types: small cell lung cancer (SCLC) and non-small cell lung cancer (NSCLC) [2]. Approximately 80–85% of all cases are diagnosed as NSCLC and about 70% of patients have locally advanced or metastatic disease at the time of diagnosis [3–5]. The overall 5-year survival rate is only 14–17% [6], due mainly, to the poor detection of lung cancer in its early stages and the ineffective treatment for advanced stages [7].

Intense inflammation is reported in NSCLC and it is significantly associated with disease progression and decreased survival of patients [8]. The inflammation caused by immune system activation is likely linked to carcinogenesis by promoting angiogenesis and proliferation of tumour cells [9,10], according to the cytokine profile in the tumour microenvironment [11–14].

Cytokines are a diverse group of small and soluble polypeptides and glycoproteins produced by various cell types, mainly by immune cells but also cancer cells, comprising interleukins (IL), interferon (IFN), chemokines, tumour necrosis factor (TNF) and growth factors [15]. Cytokines can act locally or systemically in different cell types, triggering signalling pathways responsible for cell activation, proliferation, growth, differentiation, migration and cytotoxicity [16,17]. In carcinogenesis cytokines have dual effects they can be involved in the activation of immune effector mechanisms, that limit the tumour growth, or in malignant transformation, tumour growth, invasion and metastasis [11]. Characteristic cytokine patterns have been described in different cancer patients and they are related to their diagnosis, prognosis, prediction of treatment responses [18] and survival [7].

In this study a panel of seven cytokines, frequently associated to lung cancer development, was evaluated in the plasma of patients with NSCLC and healthy controls to investigate their profile and its relationship with clinical characteristics and overall survival.

## Materials and methods

### Patients and healthy volunteers

This was a prospective, cross-sectional study, in which patients were recruited consecutively from the Chest Department of Barretos Cancer Hospital (Barretos, Brazil) from January 2013 to October 2015. For the purpose of this research, 77 patients with NSCLC confirmed by histological examinations of samples obtained during fiberoptic broncoscopy were recruited consecutively before any treatment or primary lung surgery. Pathologic stages were determined according to the criteria of World Health Organization [19]. Healthy volunteers (controls) (n = 91) were recruited from blood donors who were received at the same hospital from January to October 2015 with excellent health status at the moment of the study. The written informed consent was obtained from each volunteer upon approval of the study by Barretos Cancer Hospital Ethic Committee (number 889/ 2014) and all subjects answered a questionnaire containing socio-demographic and life style characteristics (S1 File), and authorized the use of tissue samples and clinical data for research. The median ages were 59.8 years (age range: 43–76 years) and 57.1 years (age range: 34–69 years) of the patients and the healthy volunteers, respectively.

### Blood samples

Peripheral venous blood samples (4mL) were collected in EDTA tube (BD Vacutainer, BD Biosciences, USA) from both patients and controls groups at one time point and subsequently centrifuged at 2125 g for 10 minutes at 4°C and the supernatant (plasma) frozen into microtubes at -80°C until to be used. From patients, blood was collected at pre-treatment time, more specifically prior to bronchoscopy and, from healthy individuals, blood was obtained before blood donation.

### Cytokines analysis

Plasma levels of the cytokines IL-2, IL-4, IL-6, IL-10, TNF, IFN-γ and IL-17A (human Th1/Th2/Th17 CBA kit; BD Biosciences, San Jose, CA, USA) were measured by cytometric bead assay (CBA) according to the manufacturer’s protocol. However, reaction volume was reduced to 25 μl/ sample as previously demonstrated [20]. Briefly, seven capture bead populations with distinct fluorescence intensities and coated with cytokine-specific capture antibodies were mixed together in equal volumes: 25 μL of each sample and 25 μL of PE-conjugated detection antibodies were added to 25 μL of mixed-bead populations. The mixture was incubated for 3 hours at room temperature in the dark to form sandwich complexes. The beads were then washed with wash buffer, and data acquired with a BD FACSCanto™ platform (BD Biosciences, San Jose, CA, USA). FACSDiva and FCAP Array™ software (BD Biosciences) were used for the analyses.

### Statistical analysis

The participants’ age was calculated for both groups through the T-Student test. The comparisons between socio-demographic and life style parameters were analysed using χ^2^ test or Fisher’s exact test, when indicated. The comparisons between clinical parameters and cytokines levels were analysed using T-Student test or Mann-Whitney U test. And the comparisons of cytokines levels between groups were described by mean, standard deviation, median, minimum and maximum using Mann-Whitney U Test.

The Receiver Operating Characteristic (ROC) curve was used to establish the cut-off values for the cytokines according to the event of patient’s status (died/ alive). Comparisons between the groups were analysed by χ^2^ test. The survival rate was estimated by Kaplan-Meier method and the evaluation of association between IL-6 level and survival time was estimated by log-rank test. Multivariate analyses using an adjust Cox proportional hazards model were used to identify significant independent variables. Were considered statistically significant p value <0.05.

All statistical analyses were performed with IBM SPSS Statistics software 21.0 (SPSS, Chicago, USA).

## Results

### Patients and healthy volunteers characteristics

Seventy-seven lung cancer (LC) patients and 91 control individuals were included in the study. Among patients, 45 had squamous cell carcinoma and 32 adenocarcinoma. Further, 90.5% were in TNM stage III–IV and 51.7% of tumors presented as a mass poorly differentiated. Both patients and controls were not different regarding to gender and race, nonetheless in LC patients a strong association with history of cigarette smoking is shown (p < 0.001) as well as in passive smoke exposed individuals (p < 0.001) (Table 1). To clarify, we consider active smokers those smoking at the time of interview or stopped in the last 12 months, and nonsmokers those who never smoked or who stopped for more than 12 months. As passive smokers were considered those individuals who lived with active smokers at the time of interview and / or in the past with active smokers in the home environment and / or in the workplace.

**Table 1:** Baseline characteristics of NSCLC patients and healthy subjects.

Variable	NSCLC patients	Healthy subjects	*P*
	*n* (%)	*n* (%)	
**Gender**			
Female	21 (27.3)	27 (29.7)	0.732
Male	56 (72.7)	64 (70.3)	
**Race**			
White	61 (80.3)	68 (74.7)	0.395
No white	15 (19.7)	23 (25.3)	
**Smoking history**			
No	35 (45.5)	76 (83.5)	** <0.001**
Yes	42 (54.5)	15 (16.5)	
**Passive smoke**			
No	27 (35.1)	64 (70.3)	**<0.001**
Yes	50 (64.9)	27 (29.7)	
**Histology**			
Squamous cell carcinoma	45 (58.4)	-	
Adenocarcinoma	32 (41.6)	-	
**TNM stage**			
I—II	7 (9.5)	-	
III—IV	67 (90.5)	-	
**Degree of differentiation**			
Well/ moderated	29 (48.3)	-	
Poorly	31 (51.7)	-	
Total	77	91	

NSCLC: non-small cell lung cancer; TNM stage: System based on the size and / or extent of the primary tumor (T), amount of compromised lymph nodes (N) and presence of metastases (M).

### Cytokine detection

CBA was carried out to assess the cytokine expression levels; basically classified as pro-inflammatory IL-2, IL-6, IFN-γ, TNF and IL-17A and, anti-inflammatory such as IL-4 and IL-10. Among the pro-inflammatory cytokines, IL-6 was the only one showing statistically significant difference between the groups (p < 0,001) with higher value in the patients (Table 2). No significant difference was found among the anti-inflammatory cytokines (Table 2).

**Table 2:** Cytokine expression levels between NSCLC patients and healthy subjects.

Cytokines	Groups				
	NSCLC patients		Healthy subjects		*p*
	Mean (SD) pg/mL	Median (range) pg/ mL	Mean (SD) pg/ mL	Median (range) pg/ mL	
**Pro-inflammatory**					
IL-2	2.48 (2.14)	2.07 (0–8.10)	3.06 (2.73)	2.67 (0–18.73)	0.157
IL-6	25.03 (36.40)	15.16 (0.66–231.0)	2.21 (1.30)	1.90 (0.36–7.70)	**<0.001**
IFN-γ	2.22 (7.75)	0.86 (0–68.01)	1.27 (1.45)	0.47 (0–5.72)	0.884
TNF	0.86 (0.98)	0.59 (0–4.47)	1.03 (1.98)	0.72 (0–15.17)	0.843
IL-17 A	12.76 (9.74)	10.56 (0–62.73)	12 (6.29)	10.26 (0–29.67)	0.726
**Anti-inflammatory**					
IL-4	0.92 (1.03)	0.67 (0–5.14)	1.02 (1.37)	0.89 (0–10.14)	0.692
IL-10	1.19 (1.56)	0.76 (0–9.39)	0.76 (1.05)	0.66 (0–9.84)	0.074
**Total**	**77**		**91**		

NSCLC: non-small cell lung cancer; SD: standard deviation; pg/mL: picogram/milliliter; IL: interleukin; IFN: interferon; TNF: tumor necrosis factor.

### Correlation between cytokine levels, socio-demographic and lifestyle characteristics and clinical data

The correlation between the cytokines expression with patient`s clinic-pathologic variables revealed that among all cytokines evaluated, only IL-6 and IL-17A showed statistical significant difference with socio-demographic characteristic and clinical data, respectively. Median concentration of IL-6 was statistically associated with gender, showing to be higher in male (16.67 pg/mL, range 1.44 pg/mL–34.66 pg/mL) than in female (10.06 pg/mL, range 1.44 pg/mL– 34.66 pg/mL) (p = 0.036). And, median concentration of IL-17A was associated with TNM stage, being higher levels in III-IV stage (11.82 pg/mL, range 0 pg/mL– 62.7 pg/mL) than in I-II stage (8.75 pg/mL, range 0 pg/mL– 11.8 pg/mL) (p = 0.044) (data not shown in table).

### Correlation between cytokine levels and survival

We also evaluated the association among study variables, plasma levels of cytokines and survival of NSCLC patients. However, among the variables we analyzed, only IL-6, TNM stage and degree of differentiation had a significant influence on survival rates through the analysis of Kaplan-Meier and log-rank. The time for overall survival (OS) was calculated over the interval of the dates of diagnosis and death or the date of the last information. For IL-6, the cut-off point was 8.05 pg/ml, sensitivity of 86.8% and specificity of 43.6%. Accordingly, the patients were divided into high and low plasma levels, corresponding to 55 (71.4%) and 22 (28.6%) individuals, respectively.

**Fig 1:**
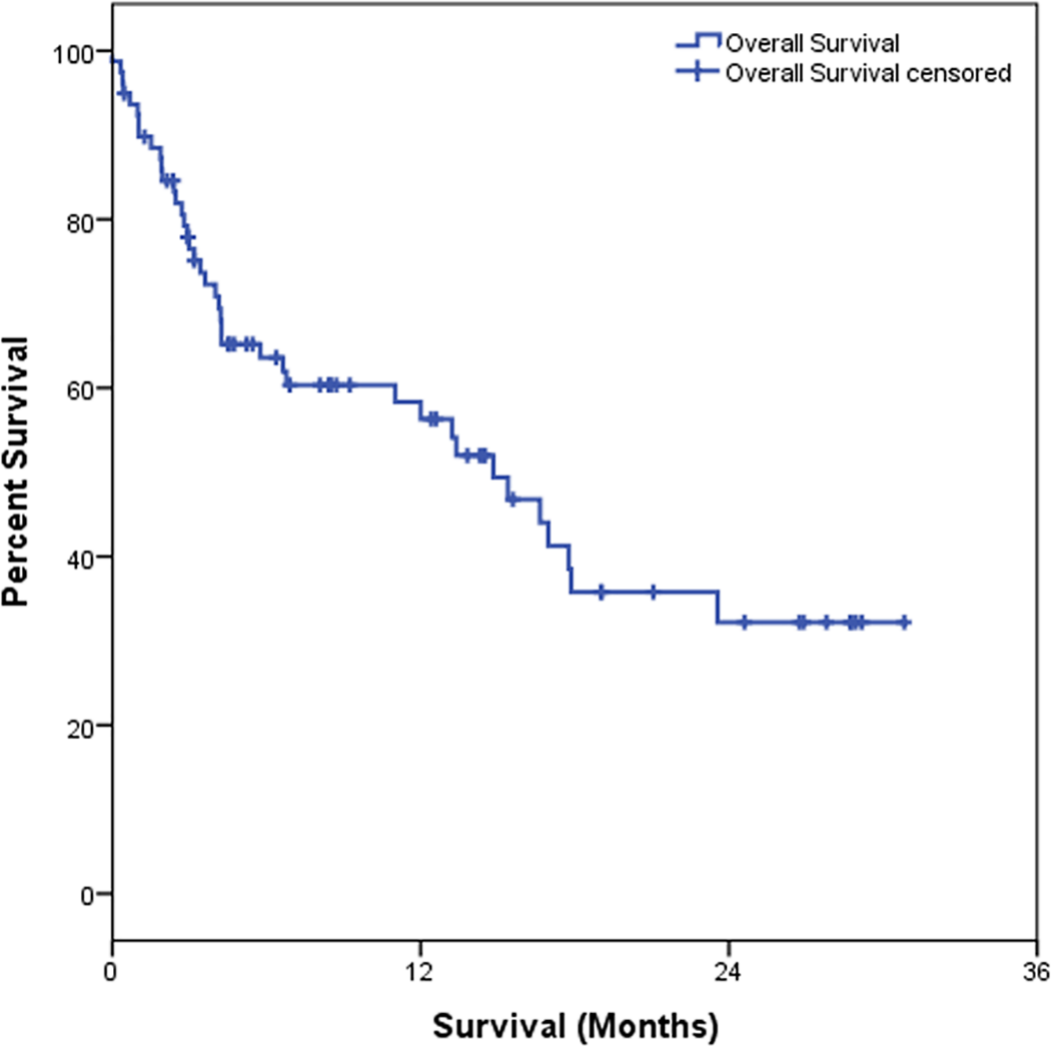
Kaplan-Meier overall survival of NSCLC patients.

**Fig 2:**
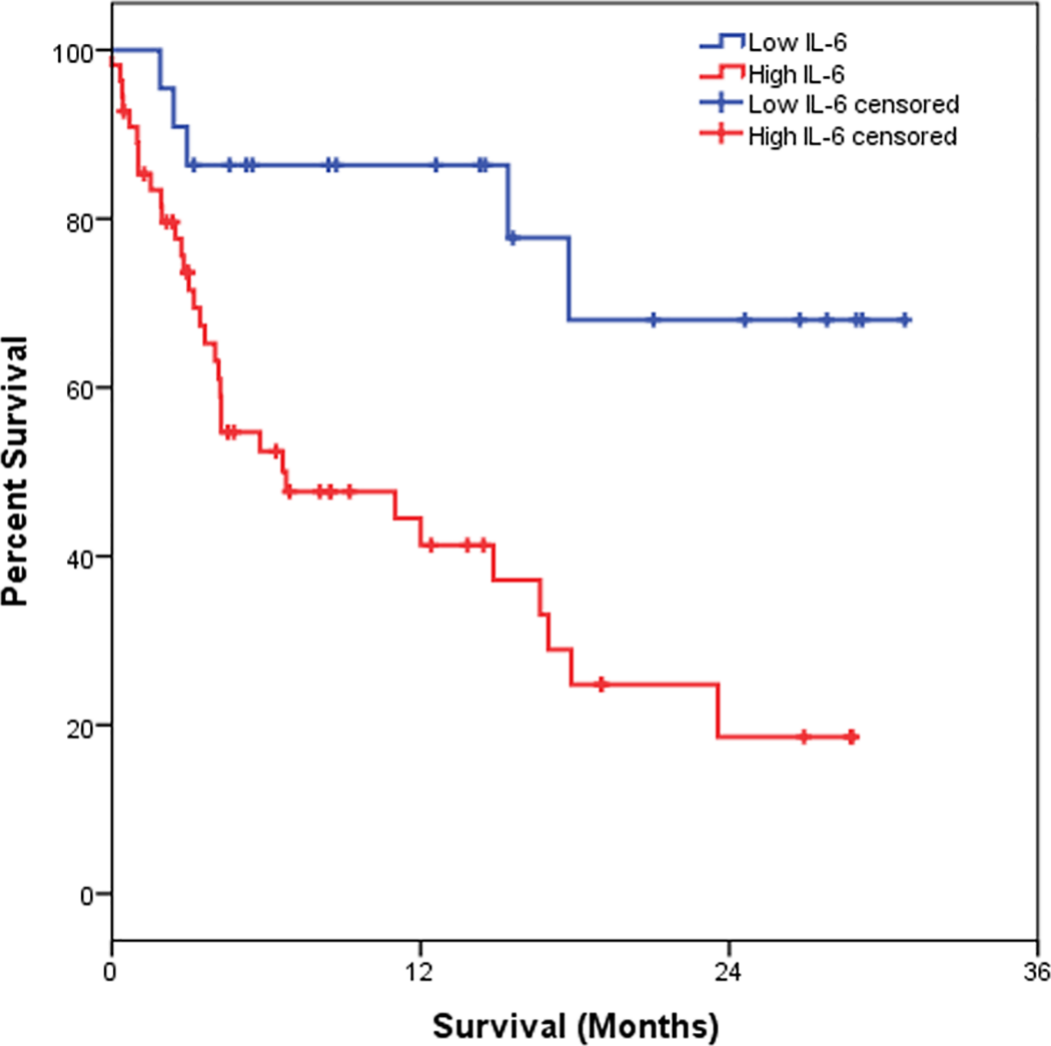
Kaplan-Meier survival estimates by stratification of serum IL-6 concentration in NSCLC patients.

**Fig 3:**
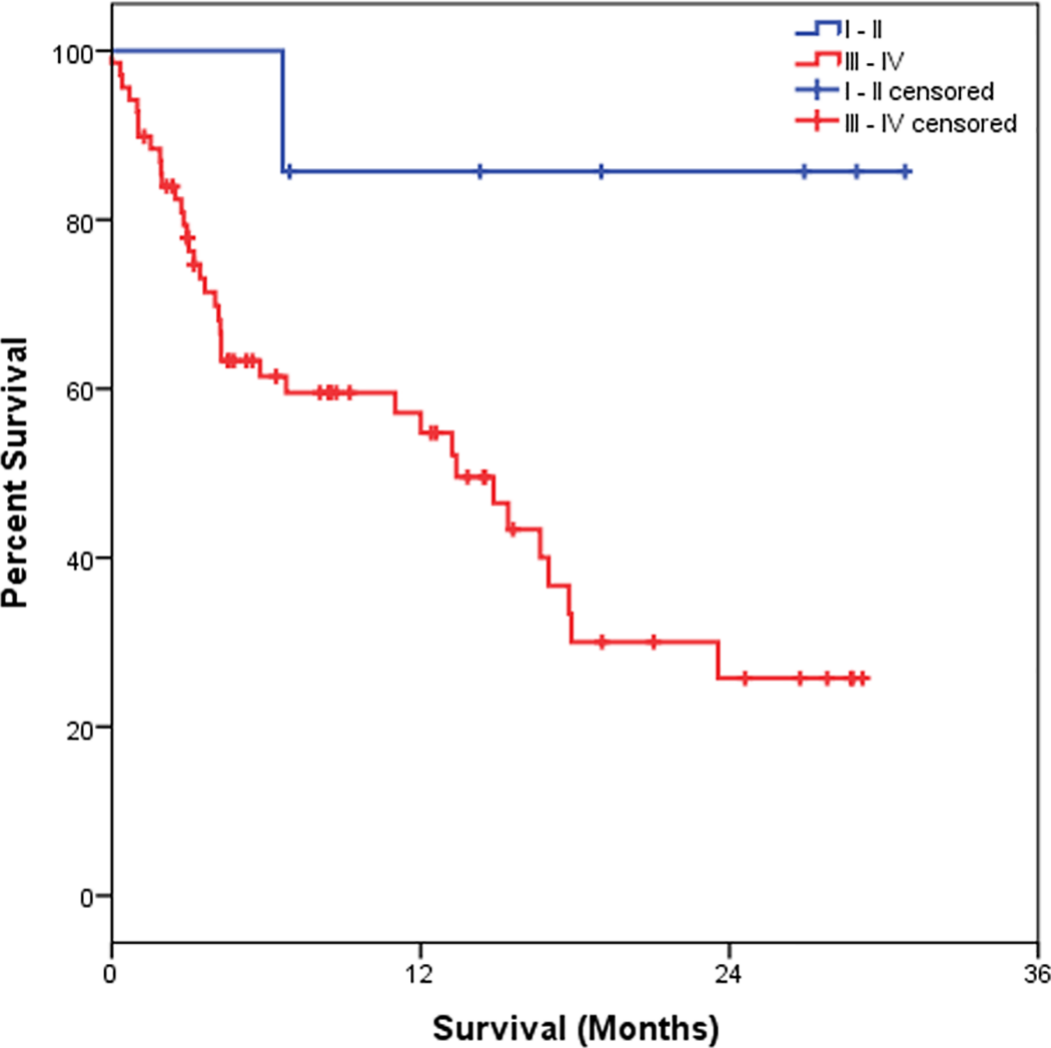
Kaplan-Meier survival estimates by stratification of TNM stage in NSCLC patients.

**Fig 4:**
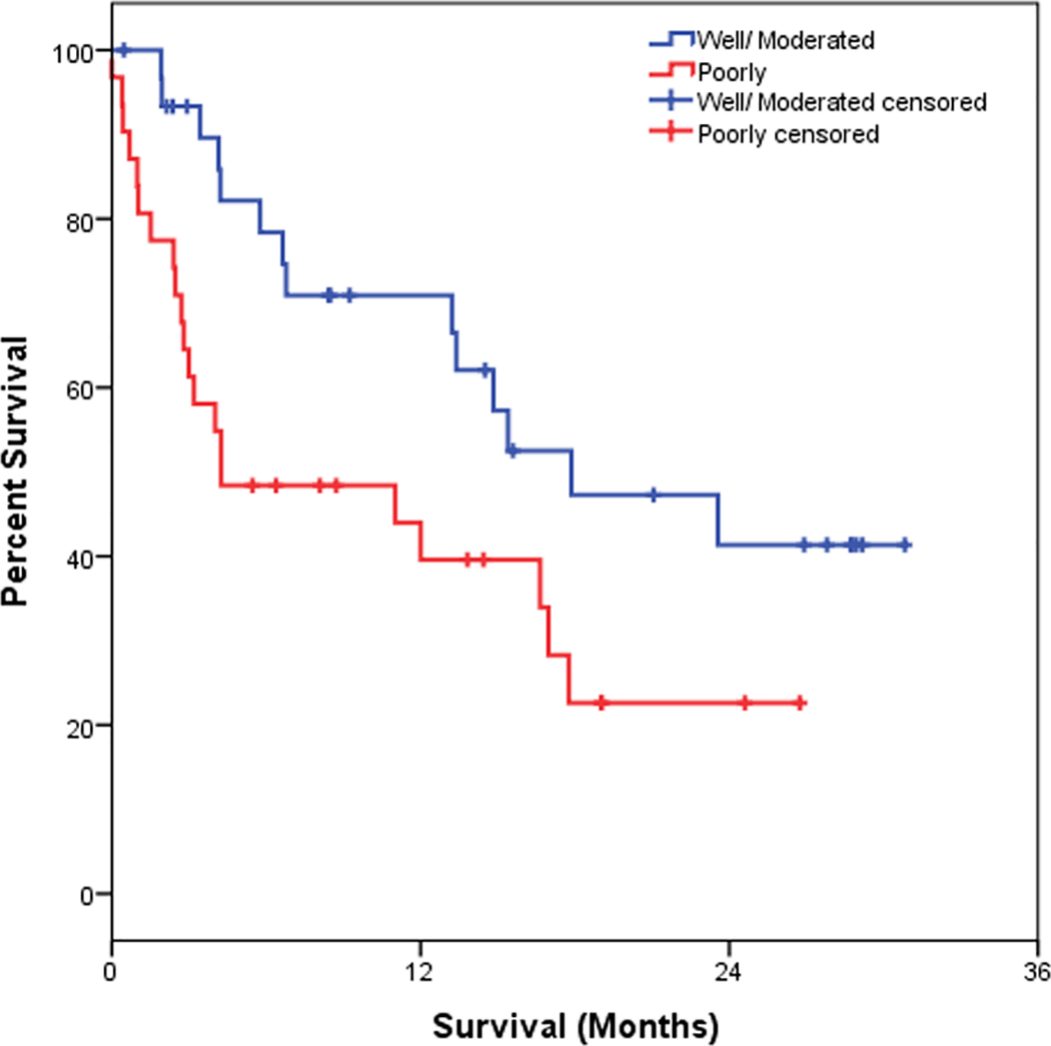
Kaplan-Meier survival estimates by stratification of degree of differentiation in NSCLC patients.

Table 3 shows that the OS estimated at 6 months was 95.5%, at 12 months was 56.3% and at 24 months was 32.2%. As for IL-6, we observed that those patients showing high IL-6 plasma level showed a significantly worse OS (p = 0.001) as compared to individuals with low IL-6 plasma levels. Considering the clinical data, we observed that patients with I–II staging had better OS at 6, 12 and 24 months, estimated at 86.4%, while those with III–IV staging presented OS estimated at 95.7% in 6 months, 54.8% in 12 months and 25.7% in 24 months (p = 0.029). For the degree of differentiation (well/ moderated and poorly), we observed a worse OS rate for patients with a poor differentiation degree when compared those with well/ moderated differentiation degree (p = 0.026). Results of Kaplan-Meier survival analyses and log-rank tests are shown below (Figs 1–4).

**Table 3:** Estimate of overall survival considering the clinical variables and plasma level of IL-6.

Variable	Categories	N° Cases	N° Deaths	Probability of survival (%)			*P*
				6 months	12 months	24 months	
**Overall survival**	-	79	40	95.0	56.3	32.2	**-**
**IL-6**	Low	22	5	86.4	86.4	68.0	**0.001**
	High	55	33	52.4	41.3	18.6	
**TNM stage**	I—II	7	1	85.7	85.7	85.7	**0.029**
	III—IV	69	37	95.7	54.8	25.7	
**Degree of differentiation**	Well/ moderated	31	14	78.4	71.0	41.4	**0.026**
	Poorly	31	21	48.4	39.6	22.6	

IL: interleukin; TNM stage: system based on the size and / or extent of the primary tumor (T), amount of compromised lymph nodes (N) and presence of metastases (M).

Additionally, the multivariate Cox regression analysis revealed that only IL-6 levels was independent prognostic factors for predicting poorer OS in NSCLC patients (Hazard ratio = 5.81, 95% CI = 1.74–19.33, p = 0.004).

## Discussion

The immune system of the lung can be represented by cells and cytokines which have different functions; under physiological conditions, the dynamics of these elements is stable and the ratio of immune cells and molecules (eg, cytokines) is maintained within normal limits, as a result there is no generation of harmful responses to the host. Specific unbalances in these physiologic immune responses, as those caused by lung cancer can serve as biomarkers and predictive factors in relation to immunotherapy [21]. Also a better understanding of the dynamics of these cells and molecules in pathologic conditions may unravel, promising strategies for the treatment of neoplasias.

Inflammatory responses play a dual role in tumor development. In some situations, they support the inhibition of tumor growth by promoting the antitumor activity of cytotoxic T cells [22], which could limit the proliferation of transformed cells (or tumor growth) and induce even elimination. On the other hand, the induction of DNA damage by free radical production in chronic inflammation [22] can contribute to create a favorable environment to tumor progression [23]. Since inflammation can be also triggered by a variety of pathogens and environmental factors [24] and once lung is vulnerable to various pathogens and gaseous pollutants the persistent exposure to these factors can trigger the production of cytokines that result a chronic inflammatory environment capable to induce cell transformation and subsequent tumor growth [24,25].

Various biological processes such as proliferation, differentiation, migration, activation, and cell growth are coordinated by cytokines, as well as the tumor development [15,26,27]. Cytokines may contribute to tumor development in, at least, two ways: stimulating cell growth and differentiation and inhibiting apoptosis of abnormal cells [9]. Some studies have shown that cytokines measured in several biological fluids, such as plasma, can reflect various diseases, including cancer [28].

When analyzed the plasma levels of cytokines between the groups we studied, only the pro-inflammatory cytokine IL-6 showed statistically significant value with increased expression in group of patients with NSCLC, and strong association with disease development. Among the functions already mentioned as being related to IL-6, stand out those involved with differentiation of T and B cells, stimulation of hematopoiesis [29] and inducing of phosphorylation of STAT3 and STAT1 transcription factors [30]. Also, IL-6 was demonstrated to be activated in many human cancers, including lung cancer [31]. In this situation, IL-6 acts directly in the prevention of apoptosis by deleting the genes involved in cell cycle and acts as an autocrine growth factor for tumors [30]. It is considered as a major pro-inflammatory cytokines related to tumor progression in NSCLC [24].

Regarding the clinical data, our study showed a correlation of peripheral level of IL-6 with only survival. However, the relationship between the high cytokine level in males was similar to those found in the literature [32]. In relation to lifestyle data, IL-6 showed high expression in smokers in both study groups, both active and passive smokers. The smoking is associated with systemic inflammation and, it is suggested that IL-6 may have a role in the inflammatory response associated with smoking [32]. When the relationship between IL-6 and active and passive smoking was analyzed only for the NSCLC group, there was no significant correlation. This suggests that the high IL-6 expression levels in NSCLC individuals are related to disease and not to smoking.

We found that IL-17A was the only cytokine evaluated that significantly correlated with advanced stages of the disease. IL-17A effects on growth and metastasis of lung cancer cells has also been explored in animal models of development of disease [33]. After measuring the size of tumor mass, the amount of cytokine at the site and density of infiltrating macrophages in cancer mass, it was observed that animals that received IL-17 exogenously exhibited greater tumor size, increased number of infiltrating macrophages in mass, greater amount of vascular endothelial cells, increased expression of VEGF (vascular endothelial growth factor), metalloproteinases 9 and 2, and TNF, as compared to the control group that received saline [33].

The role of IL-17A has also been investigated in other tumor types such as colorectal, esophageal squamous cells carcinoma, ovary and hepatocellular carcinoma. The presence of IL-17A in colorectal cancer, hepatocellular carcinoma and NSCLC is generally associated with poor prognosis, whilst the presence of IL-17A in ovarian carcinoma and esophageal squamous cells carcinoma is associated with anti-tumor response [34,35]. In general, the cytokine IL-17A produced by Th17 cells, has dual role in antitumor immunity. In one hand IL-17 can exert anti-angiogenic and apoptotic activities, on the other hand it can promote the activity of cytotoxic T cells effectors [36,37].

We also found a significant association among individuals classified as high plasma levels of IL-6 and worst probability of survival at 6, 12 and 24 months. In this line, the study of Chang et al. with 245 NSCLC patients with advanced stage of the disease, showed that high levels of IL-6 was associated with poorer response to treatment and survival in patients undergoing chemotherapy [7]. The relationship of this worst therapeutic response with deregulated expression of certain cytokines favoring resistance to treatment has been strongly suggested by other authors [38]. Despite our minute understanding of the mechanisms of resistance exerted by cytokines, it is nowadays clear that they represent potential biomarkers and therapeutic targets in cancer, requiring therefore more investigations [29].

Other studies analyzed the IL-6 expression in tumor tissue, plasma and bronchoalveolar lavage, and found a correlation with progression, resistance to anti-tumor therapies and poor survival of patients with lung cancer [7,39,40]. The association between high levels of IL-6 and lower survival was also observed in patients with renal cell carcinoma [41], chronic lymphocytic leukemia [42], gastric carcinoma [43], prostate cancer [44], gastrointestinal cancer [45] and breast cancer [46].

The high IL-6 levels detected in the circulation by us and others suggest that these cytokine may be a marker of worse prognosis for patients with advanced NSCLC or for those treated with chemotherapy [7]. Although 40% of patients with NSCLC express high concentration of IL-6, the mechanisms responsible for this correlation between IL-6 expression and poor prognosis remains to be clarified [39]. Clinical and epidemiological studies suggest a strong association between chronic inflammation and some types of cancer, including NSCLC. The increased plasma levels of cytokines can act as an independent marker of survival in patients with lung cancer. Despite the lack of understanding of the relationship of cytokines with the mechanisms of tumor progression and survival, we can not exclude that cytokines are potential circulating biomarkers, to characterize subgroups of patients and provide relevant information to new target therapies [7], with the advantage that they are easily measured and simple to obtain. Despite this, a limitation of our study is that we did not evaluate the source of Il-6 detecada nor the cell responsible for its production.

In conclusion, individuals with NSCLC exhibit high levels of IL-6 with significantly worse survival, suggesting that this cytokine may act as an independent marker of prognostic for overall survival of these patients.

## Supporting information

S1 File Questionnaire: Questionnaire with demographic information. (DOCX)

S2 File Data: Data obtained from the study population and used for the analyzes. (XLSX)
