# Supplementary material for: High systemic IL-6 is associated with worse prognosis in patients with non-small cell lung cancer
Source: PLoS One. 2017 Jul 17;12(7):e0181125. doi: 10.1371/journal.pone.0181125 (PMC5513446; doi:10.1371/journal.pone.0181125)
Supplement: S1 File — (DOCX) [file pone.0181125.s001.docx]

**Supporting Information**

**Questionnaire with demographic information**

Date interview: MM/DD/YYYY

| **1** | Study identification |  |
| --- | --- | --- |
| **2** | Name |  |
| **3** | Hospital record |  |
| **4** | Group  **1-** Case; **2-** Control |  |
| **General information** | | |
| **5** | Birth  **MM-DD-YYYY** |  |
| **6** | Gender  **1-** Female; **2-** Male |  |
| **7** | Race (skin color)?  **1-** White; **2-** Black; **3-** Brown; **4-** Asian; **5-**Native |  |
| **8** | Marital status  **1-** Single; **2-** Married; **3-** Divorced; **4-**Widowed |  |
| **9** | Level of schooling  **0-** Illiterate; **1-** Can read and write;  **2-** Incomplete elementary school; **3-** Complete elementary school;  **4-** Incomplete high school; **5-** Complete high school;  **6-** University incomlete; **7-** University complete |  |
| **Life style** | | |
| **10** | **Alcohol**  **0-** Never; **1-** Yes,even drink (if stopped in the last 12 months); **3-** Yes, only in the past |  |
| **11** | **Beer**  **0-** No; **1-** Yes; **99-**Unknown |  |
| **12** | Age of onset  **Years** |  |
| **13** | Age of ending  **Years** |  |
| **14** | Unit  **1-** Small glass – 50 ml; **2-** Glass medium – 100 ml;  **3-** Large glass – 250 ml½ or small bottle – 330 ml; **4-** Bottle – 700-750 ml;  **5-** Bottle – 1 L |  |
| **15** | How many units consume |  |
| **16** | Per  **1-** Day; **2-** Week; **3-**Month; **4-**Year |  |
| **17** | **Wine**  **0-** No; **1-** Yes; **99-**Unknown |  |
| **18** | Age of onset  **Years** |  |
| **19** | Age of ending  **Years** |  |
| **20** | Unit  **1-** Small glass – 50 ml; **2-** Glass medium – 100 ml;  **3-** Large glass – 250 ml½ or small bottle – 330 ml; **4-** Bottle – 700-750 ml;  **5-** Bottle – 1 L |  |
| **21** | How many units consume |  |
| **22** | Per  **1-** Day; **2-** Week; **3-**Month; **4-**Year |  |
| **23** | **"Cachaça"**  **0-** No; **1-** Yes; **99-**Unknown |  |
| **24** | Age of onset  **Years** |  |
| **25** | Age of ending  **Years** |  |
| **26** | Unit  **1-** Small glass – 50 ml; **2-** Glass medium – 100 ml;  **3-** Large glass – 250 ml½ or small bottle – 330 ml; **4-** Bottle – 700-750 ml;  **5-** Bottle – 1 L |  |
| **27** | How many units consume |  |
| **28** | Per  **1-** Day; **2-** Week; **3-**Month; **4-**Year |  |
| **29** | **Tobacco**  **0-** No; **1-** Yes, currently, **2-** Yes, in the past |  |
| **30** | **filter cigarette**  **0-** No; **1-** Yes; **99-**Unknown |  |
| **31** | Number per day  **Number** |  |
| **32** | Age of onset  **Years** |  |
| **33** | Age of ending  **Years** |  |
| **34** | **Cigarettes without filter**  **0-** No; **1-** Yes; **99-**Unknown |  |
| **35** | Number per day  **Number** |  |
| **36** | Age of onset  **Years** |  |
| **37** | Age of ending  **Years** |  |
| **38** | **Pipe**  **0-** No; **1-** Yes; **99-**Unknown |  |
| **39** | Number per day  **Number** |  |
| **40** | Age of onset  **Years** |  |
| **41** | Age of ending  **Years** |  |
| **42** | **Cigar**  **0-** No; **1-** Yes; **99-**Unknown |  |
| **43** | Number per day  **Number** |  |
| **44** | Age of sunset  **Years** |  |
| **45** | Age of ending  **Years** |  |
| **PASSIVE EXPOSURE TO CIGARETTE SMOKE** | | |
| **46** | Have you alerady been married (or lived together) with a smoker?  **0-** No; **1-** Yes; **2-** I do not remember; **99-**Unknown |  |
| **47** | Age when spouse has begun |  |
| **48** | Age when spouse has stopped |  |
| **49** | Number of hours that you were exposed during the week |  |
| **50** | Number of hours that you were exposed during the weekend |  |
| **51** | Have you already worked in a placeclose where people smoked?  **0-** No; **1-** Yes; **2-** I do not remember; **99-**Unknown |  |
| **52** | Age when you started working with smokers |  |
| **53** | Age when you stopped working with smokers |  |
| **54** | Number of hours/day that you were exposed |  |
| **55** | Number of hours that smoked in your presence |  |
| **56** | When you were a child, your parents smoked?  **0-** No; **1-** Yes; **2-** I do not remember; **99-**Unknown |  |
| **57** | How long were you exposed?  **Years** |  |
